# Dimethyl Fumarate vs. Monomethyl Fumarate: Unresolved Pharmacologic Issues

**DOI:** 10.3390/pharmaceutics17121506

**Published:** 2025-11-22

**Authors:** Jana Kopincova, Iveta Bernatova

**Affiliations:** Centre of Experimental Medicine, Slovak Academy of Sciences, Institute of Normal and Pathological Physiology, 813 71 Bratislava, Slovakia

**Keywords:** DMF, MMF, carboxylesterase, NRF2, glutathione, KEAP1, repurposing

## Abstract

Dimethyl fumarate (DMF) has established a significant position among therapies for multiple sclerosis and psoriasis and is now being investigated for repurposing to many other non-malignant diseases. Despite decades of preclinical research, some issues about its pharmacology remain unresolved, with ongoing debate over which of the methyl esters of fumarate, whether DMF or monomethyl fumarate (MMF), is the active ingredient. It is generally accepted that DMF undergoes enzymatic hydrolysis to MMF and methanol. However, there is disagreement regarding its exact site, its extent, and the responsible enzyme(s). The enzymatic mechanisms, particularly the roles of carboxylesterases-1 and 2, vary across tissues and species, complicating the translation of in vitro and in vivo preclinical findings into clinical practice. In addition, the impact of DMF and MMF is often not clearly distinguishable and sometimes overlaps, making the true molecular mediators of therapeutic and side effects unclear. Thus, the interpretation of some results obtained in studies is inconsistent because of interchanging of in vitro and in vivo observed features of fumarate esters: while DMF demonstrates rapid and strong effects in cell culture studies, including nuclear factor erythroid 2-related factor 2 (NRF2) function activation and glutathione depletion, these observations may not exactly reflect systemic pharmacology and physiology dominated by MMF. Moreover, methanol, the co-product of DMF metabolism, may contribute to the observed DMF effects through increased production of reactive oxygen species, which could result in activation of NRF2-dependent mechanisms. This review highlights specific unresolved issues in DMF metabolism, which are sometimes overlooked.

## 1. Introduction

Deeper insight into the pharmacology of approved drugs enhances the possibility of their successful repurposing for diseases that share common etiological and pathomechanistic characteristics with the original on-label indications. Dimethyl fumarate (DMF), formerly used as a fungicide, and currently approved for the treatment of psoriasis and multiple sclerosis, is certainly no exception [1,2].

As an activator of the essential antioxidant protection of the cell, the nuclear factor erythroid 2-related factor 2 (NRF2) pathway, DMF could logically be beneficial in any condition rooted in redox imbalance or oxidative stress [3,4]. Additionally, its ability to inhibit nuclear factor κB (NF-κB) p65 phosphorylation and suppress NLRP3 inflammasome makes DMF a promising candidate for therapeutic repurposing in diseases involving inflammatory cascades [5,6,7,8,9,10].

One mechanism of action attributed to DMF is the activation of hydroxycarboxylic acid receptor 2 (HCAR2), which has been linked to both therapeutic effects and adverse events—particularly skin flushing, that along with gastrointestinal discomfort, represents one of the most common side effects of DMF therapy [11,12]. DMF is frequently cited to be an agonist or ligand of HCAR2 [13,14,15]. However, HCAR2 is not activated by DMF itself; no such interaction has been demonstrated—on the contrary, DMF has been shown to be inactive in this context [16]. Instead, HCAR2 is activated by monomethyl fumarate (MMF), the primary metabolite of DMF, which is formed via esterase-mediated hydrolysis of DMF, and accompanied by the release of methanol [17,18,19].

And this leads to the core of this review. According to current pharmacokinetic understanding, DMF undergoes rapid pre-systemic hydrolysis following oral administration—partly within the intestinal lumen, and predominantly within enterocytes or during first-pass hepatic metabolism [19,20,21,22,23,24]. In the peripheral blood of DMF-treated patients, intact DMF is not detectable, whereas MMF, its active metabolite, reaches peak plasma concentrations approximately 2–2.5 h post-dose [2,25]. It appears that most—if not all—clinical effects are mediated by MMF, given that DMF may not reach target tissues within the body at all.

Despite these findings, DMF remains widely used in in vitro studies, and the resulting data are typically understood as indicative of its therapeutic mechanisms in humans. A large portion of researchers in both preclinical and clinical sciences maintain the perspective that considers DMF as the primary active ingredient, with MMF functioning as its (active) metabolite. The statement “Monomethyl fumarate (MMF) and its prodrug dimethyl fumarate (DMF) are currently the most widely used agents for the treatment of multiple sclerosis.” [11] is still not quite common.

However, another scientific view regards DMF as a prodrug—essentially a “methyl ester of MMF”—with MMF representing the pharmacologically active moiety responsible for the therapeutic effect. In this view, emphasis is given to the fact that mechanisms observed in vitro may not be physiologically relevant, as DMF is hydrolysed to MMF (and methanol) before reaching systemic circulation.

Between these perspectives lies a body of literature that, for instance, acknowledges that DMF is completely hydrolysed in the intestinal mucosa, yet still includes figures depicting DMF binding to Kelch-like ECH-associated protein 1 (KEAP1) in specialized cells. Or examines DMF metabolism in differentiated tissue cells and then states that only MMF is detectable in the portal vein following oral administration. In reviews focused on DMF, the attempt to cite as many studies as possible sometimes results in the inclusion of conflicting data without critical consideration, while in other cases, authors refrain from distinguishing between DMF and MMF and perceive them as interchangeable molecules or simply as “two forms” of the same compound.

However, MMF displays markedly different pharmacokinetic and pharmacodynamic properties compared to DMF (Table 1), and, unlike DMF, has failed to demonstrate efficacy in numerous in vitro models [26,27].

As previously noted, successful drug repurposing assumes a thorough understanding of pharmacokinetics (PK) and pharmacodynamics (PD)—and in the case of DMF, even some fundamental questions remain unresolved. This review does not aim to map all gaps and inconsistencies in our knowledge of DMF and MMF pharmacology. Rather, it seeks to highlight specific unresolved—or even unexplored—issues, and to draw attention to facts that are often overlooked or considered unimportant.

## 2. Brief Overview—Basic Features of DMF and MMF

Dimethyl fumarate (IUPAC name: dimethyl (E)-but-2-enedioate) is the dimethyl ester of fumaric acid, an α,β-unsaturated carboxylic ester with moderate lipophilicity, resulting in relatively low solubility in water (1.6 mg/mL) and higher solubility in organic solvents such as dimethyl sulfoxide or methanol (29 and 30–36 mg/mL, respectively) [28]. Its basic chemical characteristics include a non-polar molecule with high cell permeability, possessing two methyl groups at each end, and an unsaturated double bond. Similar to fumarate itself, the double bond of DMF central trans-alkene group enables spontaneous covalent interaction with nucleophilic groups (such as thiols or cysteine residues in proteins) via a non-enzymatic Michael addition [32,33,34]. The resulting modification (including the oxidation of sulfhydryl residues), known as succination, is considered one of the major mechanisms through which fumarate and its derivatives influence cellular processes, notably by reducing glutathione (GSH) bioavailability through irreversible adduct formation [32,34,40]. The effect of Michael acceptors, and namely DMF, on protein expression of heme oxygenase-1 (HO-1) through antioxidant-responsive elements (ARE) was mentioned as early as 1995 [41], while NRF2 and its repressor KEAP1 were added to this scheme a few years later [42,43]. Nowadays, activation of the NRF2 pathway by modifying cysteine residues on the KEAP1 protein is generally accepted as the main “on-target” effect of DMF [44,45].

In clinical practice, DMF is used for the treatment of multiple sclerosis (MS) and psoriasis, primarily in the form of delayed-release capsules or enteric-coated tablets containing DMF alone or in combination with salts of monoethyl fumarate (MEF), marketed under the brand names Fumaderm^®^ (for psoriasis, since 1994), Tecfidera^®^ (for MS, since 2013), and Skilarence^®^ (for psoriasis, since 2017) [19,46]. Alternative routes of administration are being investigated, including a particularly interesting recent study on intranasal administration of hyaluronic acid-modified lipid–polymer hybrid nanoparticles loaded with DMF, which were shown to reach the cerebrospinal fluid without entering systemic circulation [30,47].

Following the discovery that the active ingredient is actually the DMF metabolite MMF, pharmaceutical formulations were also developed using the MMF precursor diroximel fumarate as Vumerity^®^ (for MS, since 2019), the latest one, tegomil fumarate as Riulvy (for MS, since 2025), and MMF itself as Bafiertam™ (for MS, since 2020), with the aim to lower undesirable side effects of DMF, such as gastrointestinal events and flushing [2,48,49]. Another derivative, tepilamide fumarate, has recently proven effective in the Phase IIb clinical study AFFIRM for the treatment of psoriasis (NCT03421197, clinicaltrials.gov), and isosorbide di-(methyl fumarate) is under investigation [50,51]. Dosage forms, strengths and dosing are summarized in Table 2.

Monomethyl fumarate (IUPAC name: (E)-4-methoxy-4-oxobut-2-enoic acid), sometimes referred to as methyl hydrogen fumarate, is the monomethyl ester of fumaric acid and differs from DMF in several important chemical properties. The most notable distinction lies in the polarity of MMF, which increases its water solubility while reducing its ability to permeate biological membranes. This is manifested in prolonged *t_max_* (time to reach maximum observed plasma concentration, *C_max_*) of MMF by approximately 1.5 h compared to the reference DMF (4.03 h and 2.5 h, respectively), and by approximately 1 h compared to diroximel fumarate (4.0 h and 3.0 h, respectively), after oral administration of Bafiertam™, Tecfidera^®^ and Vumerity^®^ to healthy volunteers [48,58].

The polarity of MMF also influences its other properties—for example, the affinity of carboxylesterases (CESs), whose structural conformation is generally more suitable for binding non-polar (hydrophobic) molecules such as DMF [23,59]. Poor affinity of CESs towards MMF might be reflected in the lower interindividual variability observed in MMF pharmacokinetic parameters following Bafiertam^TM^ administration, when compared to Tecfidera^®^ [58], where MMF delivery depends on carboxylesterase activity—a process known to exhibit substantial interindividual variability [60].

Although the α,β-unsaturated double bond—just as in DMF—allows for Michael addition, the formation of GSH adducts proceeds significantly more slowly with MMF than with DMF [37]. This may preserve the MMF molecule from binding to intracellular GSH during intestinal absorption [22] and may be one of the reasons why a 190 mg dose of MMF is bioequivalent (i.e., produces equivalent plasma exposures of MMF) to 240 mg of DMF [58].

The differences between DMF and MMF are so substantial that researchers have warned against their interchangeable use in the literature [61] (Table 1). Nevertheless, it appears that the characteristics of one compound are still sometimes incorrectly attributed to the other when results obtained on isolated cell lines are translated to whole-body physiology.

## 3. Different Experimental Approaches—Different Findings

If one intends to investigate or compare the effects or properties of DMF and MMF, several fundamentally different approaches are available. This influences the interpretation of results, so one should always be aware of the study type—especially its limitations—and understand whether the described experimental conditions are relevant within the context of human physiology.

To explore chemical properties of fumarate derivatives—such as solubility, chemical stability at varying pH or temperature, stability in the presence of enzymes, or rate of molecular interactions (e.g., adduct formation via the Michael addition)—in vitro “test-tube” methods are typically used. In these experiments, either DMF or MMF is dissolved in solution while environmental conditions (temperature, pH) are altered, or substances with which fumarates may react are varied. Obviously, the results incorporate time course and concentration ([28,29,62,63] and others). These measurements are sometimes referred to as “extracellular” or cell-independent assays, as they do not involve living cells, and are usable for the investigation of both PK and PD properties.

A methodological intermediate between purely chemical reactions and cell culture-based studies is the analysis of DMF and MMF activity in biological fluids such as plasma, whole blood, or intestinal perfusates (e.g., [23,29,64]). These experiments also capture both PK and PD aspects, such as enzymatic hydrolysis or binding with biological complexes, without introducing the complexity of whole organisms.

In vitro studies using cell cultures typically involve incubation of defined, specific cell lines of different types with DMF or MMF at varying concentrations and time intervals. The readouts can include metabolic changes, e.g., GSH adduct formation, or expression changes in signalling pathways such as NRF2, NF-κB, or their direct targets like HO-1 or cytokine production and generally contribute to mechanistic understanding ([5,6,7,61,65], and many others). However, these results do not necessarily translate across all cell types, as variations in membrane channels, enzymatic composition, or internal reserves can be substantial—a point that will be further discussed, as it often contributes to discrepancies in the evaluation and interpretation of results.

Ex vivo measurements represent an intermediate step closer to physiological relevance, such as experiments conducted on tissue fragments—for example, liver homogenates or intestinal mucosa samples (as studied by [23]). These allow for the observation of metabolic or transport properties of DMF and MMF within the complexity of tissue, but in the absence of systemic circulation; however, they are now rather rare.

In vivo approaches involve oral administration of fumarate esters, either via gavage or in solution, followed by monitoring of drug/metabolite concentrations, physiological effects, gene expression, or excretion patterns, in target organs, blood, or other tissues in animal models, or by assessing available parameters after drug administration in humans. This model is the most complex and enables comprehensive PK and PD studies, while reflecting the complexity of the whole body ([3,22,48,58,66], and others). Care must be taken when translating results from animal experiments to the human body due to distinct enzymatic and metabolic parameters.

Finally, clinical or retrospective studies examine data from treated patients to assess dose–response relationship, therapeutic efficacy, safety or to analyze adverse events such as gastrointestinal intolerance or leukopenia, providing crucial insights into long-term outcomes in real-world clinical settings (e.g., CONFIRM—NCT00451451; AFFIRM—NCT03421197; ASSURE—NCT02090413, and others).

**Figure 1:**
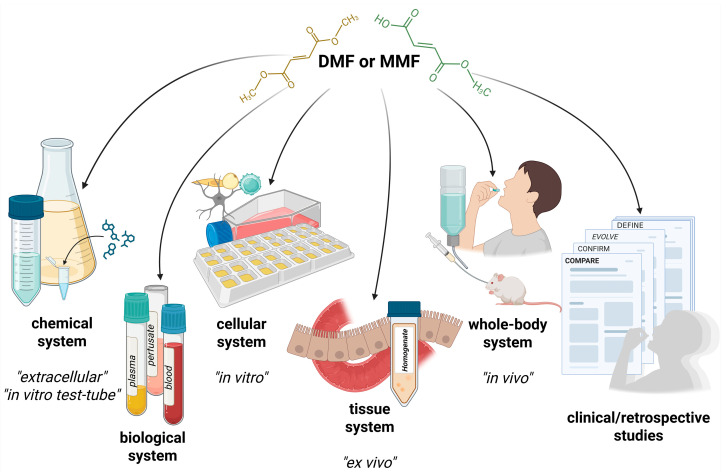
Various experimental approaches for investigating dimethyl fumarate and monomethyl fumarate effects. Abbreviations: DMF—dimethyl fumarate, MMF—monomethyl fumarate. Created in BioRender. Kopincova, J. (2025) https://BioRender.com/zp4szt8, accessed on 18 November 2025.

Despite the availability of numerous research approaches (Figure 1), and the long-term clinical use of DMF, as well as extensive high-level investigations across various physiological and molecular biology domains, the pharmacodynamics of DMF and MMF remain incompletely understood. The most significant point of debate is still unanswered: which one is the active ingredient?

## 4. Issue 1: DMF vs. MMF—Which One Is Acting?

As a non-polar, lipophilic molecule, DMF is predisposed to cross the lipid-rich environment of cellular membranes via passive diffusion without any restrictions. Its cellular permeability is much greater compared to the more polar MMF—it was reported to be up to ten times higher than that of MMF, regardless of transport direction, as shown in transport studies in 2003 by Werdenberg et al., using Caco-2 cell monolayers [23]. Perhaps for this reason, it was long thought that DMF passes freely into systemic circulation or even directly into the tissues, and reaches the site of its action more rapidly and at higher concentrations than the corresponding monoester [14,23,67].

Yet, in the same study by Werdenberg et al., when Caco-2 cells were replaced by excised porcine intestinal mucosa, no measurable amount of DMF was detected on the opposite side of the intestinal layer, presumably due to its rapid hydrolysis to MMF within just 10 min during transcellular passage through enterocytes [23]. Esterase-mediated conversion of DMF to MMF was confirmed shortly thereafter by its rapid clearance in human serum, and even faster in whole blood [29].

According to Werdenberg’s observations, MMF had become widely accepted as the active ingredient of orally administered DMF and was, therefore, recommended for use in any in vitro studies. [23,62,68,69,70]. Supporting this, there was growing evidence for the absence of DMF in systemic circulation following its oral administration, while its metabolite MMF exhibits a clearly determinable plasma peak: *t_max_* of 210 min and *C_max_* of 11.2 µM (~1.46 mg/L) after administration of Fumaderm^®^, *t_max_* of 178 min, *C_max_* of 0.8 mg/L after Fumaraat 120^®^, and *t_max_* of 120–150 min with *C_max_* of 1.87 mg/L following Tecfidera^®^ administration [71,72,73].

These findings were explained mainly by two processes: the rapid and complete spontaneous and esterase-mediated metabolism of DMF to MMF, and its binding to nucleophilic molecules such as GSH, leading to the formation of conjugates. GSH-DMF conjugation, in test-tube experiments, was found to proceed at a rate approximately 30 times faster than that of MMF [37]. Both of these reactions appear to occur as early as in the intestinal mucosa (e.g., [63,64]; for a detailed review, please see [74]), or at the latest during its passage to the liver, a process referred to as first-pass metabolism or pre-systemic hydrolysis [21,71,75].

However, with the aim of better understanding the molecular mechanisms underlying the effects of DMF treatment, studies in which isolated cell lines were incubated separately with DMF or MMF have been conducted. Many of them revealed a pronounced effect of DMF, but not MMF, on various cell types, such as human T cells, adipocytes, neurons, and astrocytes [26,27,65,76,77]. A compelling example comes from a detailed study by Gillard et al., who compared the effects of DMF and MMF on the complex protein expression profiles of various cell types, including primary human endothelial cells, peripheral blood mononuclear cells, primary human fibroblasts, and primary human keratinocytes following a 30 min preincubation. While DMF and MMF exhibited broadly similar profiles, DMF showed significantly greater potency—inducing changes at much lower doses than MMF [61].

This could have many explanations, and at least two of them seem plausible. First, the approximately 10-fold lower permeability of MMF compared to DMF, which results in vastly different intracellular concentration after 30 min. Second, the 30-fold higher ability of DMF to form GSH-DMF adducts, which results in altered redox balance [22,37,63]. Regarding this, any effect of DMF after short-term preincubation—or any effect potentially mediated by GSH depletion (e.g., induction of apoptosis, increase in HO-1 expression, etc. [22])—is expected to be significantly more pronounced in vitro when compared to MMF.

Nevertheless, the limited efficacy of MMF observed in isolated in vitro cell studies, such as that of Gillard et al., raised doubts about its therapeutic relevance in vivo and prompted efforts to investigate whether DMF itself might exert pharmacological activity—even in the absence of detectable systemic levels. To support this possibility, several hypotheses were proposed, including incomplete hydrolysis or the idea that DMF may bypass enterocytic metabolism and reach the lymphatic system directly due to its high lipophilicity [61,67,71,78]. Anyway, the presence of GSH-DMF adduct degradation products in human urine [79] keeps the question of whether DMF undergoes complete or incomplete first-pass metabolism open—and the debate ongoing.

Due to persistent concerns regarding the truly active ingredient, more recent publications have occasionally adopted the joint term “DMF/MMF” when describing the in vivo effects of fumarates at the molecular level [80,81]. This terminology can be misleading in certain cases, particularly given the distinct characteristics of these fumarate derivatives in terms of receptor binding.

Although the use of methylated forms of active compounds is common in pharmaceutical practice and does not affect their systemic classification, interchanging DMF and MMF in the context of in vitro and in vivo effects can lead to confusion in comprehension.

## 5. Issue 2: DMF After Oral Administration

The fate of DMF in the human body following oral administration remains insufficiently understood, and significant differences in pharmacokinetics exist between humans and experimental animals.

In human medicine, fumarates are generally administered orally via enteric-coated tablets or delayed-release capsules. This protects the compounds from gastric processing and serves as a preventive measure against gastric ulceration [19]. Unlike in humans, experimental animals generally receive DMF via chow, colloidal suspension, gavage, or drinking solution. In all of these cases, enteric protection is absent, and DMF is exposed to the physiological environment immediately upon entry into the body.

As noted, the main feature of the DMF molecule is its high membrane permeability. Despite this property, it is plausible that unprotected DMF exhibits limited absorption in the upper gastrointestinal tract. The oral phase, swallowing, and oesophageal transit each typically last no more than 10 s, which may be insufficient for effective DMF contact with epithelial surfaces.

In fact, the stomach is the first site where DMF may reside for a longer period and potentially interact with epithelial cells—particularly during the lag phase of gastric emptying [82]. The acidity of gastric juice and the presence of digestive enzymes therefore raise questions about the chemical stability of DMF in this environment and, consequently, the accuracy of in vivo animal experiments using the oral route of administration.

Under acidic conditions, DMF has been reported as both chemically stable [29] and chemically labile [83,84]. The discrepancy between these findings highlights the importance of critical evaluation of both methodology and interpretation of observed results. Litjens et al., using simple test-tube incubation in 0.1 N HCl at 37 °C, observed no degradation of DMF, MMF, or monoethyl fumarate after 6 h, simulating gastric conditions [29]. In contrast, other authors reported DMF degradation in 0.1 N HCl at 100 °C and 80 °C, respectively, and concluded that DMF is “labile”, although only 11% degradation was observed at 40 °C [83,84]. Thus, it appears that DMF is chemically stable in the acidic environment. However, its stability in the presence of gastric digestive enzymes such as pepsinogen or gastric lipase remains to be elucidated, as there are limited data on whether DMF serves as a substrate for these enzymes.

There is some possibility that uncoated DMF may penetrate the gastric mucosa and undergo metabolic conversion within cells. The stomach is known to absorb certain fat-soluble compounds, such as ethanol and aspirin [85,86,87]. In the absence of an enteric coating, a small amount of DMF might be absorbed in the gastric lining and bound to GSH or enzymatically hydrolysed to MMF and methanol by CESs—which are present here similarly to the intestinal mucosa. Methanol has previously been overlooked in this context, despite its own significant actions, which will be mentioned later.

The pharmacokinetics of orally administered DMF is influenced by simultaneous food intake, particularly of meals high in fat, which in humans can delay MMF *t_max_* by up to several hours without affecting the overall area under the concentration-time curve (AUC) [1,72]. Co-administration with food is also one of the recommended strategies to manage early-onset adverse events such as nausea, vomiting, and abdominal pain, as described in multiple reviews focusing on side effects associated with the initiation of DMF therapy [88,89].

Upon entering the duodenum and subsequent sections of the small intestine, the enteric coating of DMF tablets—composed of methacrylic acid copolymers—becomes ionized and dissolves due to the rise in pH. In this way, the coated DMF is released into the intestinal lumen. The change in pH also initiates slow, spontaneous hydrolysis of unprotected DMF, as described previously [19,29]. In addition, various esterases have been reported to be present in pancreatic and intestinal fluids, which are capable of cleaving the DMF molecule, although specific enzymes have not been identified [23,90].

Though DMF hydrolysis likely occurs to some extent already in the lumen of the small intestine, a substantial portion of DMF (as well as other lipophilic precursors of MMF) freely crosses into enterocytes and is subjected to metabolism within the intestinal mucosa. In contrast, MMF itself exhibits minimal passive membrane permeability. Its absorption depends probably on Na^+^/dicarboxylate transporter 1 (NaDC1, known also as SLC13A2), which is highly expressed throughout mammalian intestine (and other tissues) and functions to absorb citric acid cycle intermediates from the intestinal lumen [91]. The need of a specific transporter delays MMF *t_max_* after oral administration in comparison to its lipophilic precursors, however, the unnecessity of esterases for its activation results in higher plasma *C_max_* and AUC, and also independence of co-administered food [48]. Moreover, the presence of HCAR2 (also known as GPR109A or niacin receptors) on the luminal site of enterocytes, which are also largely expressed throughout the intestine and colon [92], allows MMF to exert its anti-inflammatory effects already at the intestinal level, as, for example, in the restoration of the intestinal barrier in Parkinson’s disease model [93]. The ligation of MMF to intestinal HCAR2 should receive more attention due to their important role in gut–brain axis [94].

According to findings in rats, upon entering to the enterocytes, DMF undergoes reactions with above mentioned GSH, and likely also with other thiol-containing molecules. Enzymatic hydrolysis to MMF and methanol is also presumed to occur at this level by some authors [22]. The initial processing of DMF in the intestinal mucosa—likely involving the intensive formation of irreversible adducts with sulfur-containing residues—may account for the observed discrepancy between the plasma MMF *C_max_* achieved after direct administration of DMF solution into the rat intestine [22], and similar plasma MMF *C_max_* after intravenous administration of MMF; where 20 mg/kg DMF dose in intestine solution led to MMF *C_max_* ~61.67 µg/mL comparable with MMF *C_max_* ~55 μg/mL study after the intravenous infusion of MMF equivalent to only 2 mg/kg of DMF [30,47]

Conversely, alcohol ingestion prior to oral DMF administration has been shown to cause significant increase in the plasma DMF *C_max_* and AUC, accompanied by pronounced decrease in both MMF *C_max_* and AUC in plasma and brain of rats [24].

The exact mechanisms to which DMF is subjected in the stomach, enterocytes, portal vein or liver remain unclear. In all of these compartments, esterase-mediated hydrolysis of DMF appears to occur. However, despite seeming familiar to those working in the field, this aspect of DMF metabolism remains largely unresolved.

## 6. Issue 3: Carboxylesterase-Mediated Hydrolysis of DMF—Familiar and Unknown

The statement that “DMF is rapidly hydrolysed by esterases” has been repeatedly cited over the years, sometimes accompanied by descriptors such as “intestinal”, “plasma”, or even “methyl” esterases—without clearly identifying the specific enzymes responsible [27,63,72,75,88].

However, the question of which esterases—and in which organs or cell types—mediate the hydrolysis of DMF remains unresolved. The lack of definitive knowledge regarding the specific enzymes responsible for DMF cleavage is often addressed by attributing the process to “nonspecific esterases”, whose presence or activity cannot be conclusively confirmed or excluded. On the other hand, it has been consistently demonstrated that DMF is hydrolysed by a group of serine hydrolases known as carboxylesterases.

Carboxylesterases (EC 3.1.1.1) are enzymes that hydrolyse ester bonds between an alcohol moiety and a carbonyl-containing acyl group—which applies to DMF. In humans, CESs are primarily located in the membrane of the endoplasmic reticulum, and their expression exhibits considerable interindividual variability [60]. The secreted form of CES has only been identified in rodent plasma [95].

These glycoproteins, composed of approximately 550 amino acid residues, are typically classified into five or six groups based on their amino acid sequences. Among them, the most extensively characterised are members of the carboxylesterase-1 (CES1) and carboxylesterase-2 (CES2) families, which serve as classic xenobiotic-metabolizing enzymes involved in the metabolism of a variety of ester-containing drugs, prodrugs, and environmental toxins [96,97].

As with their distribution, CES1 and CES2 also differ in their substrate specificities. In the intestinal mucosa, the dominant carboxylesterase is CES2 and its activity appears to be nearly constant along the human jejunum and ileum. It also dominates in the adrenal gland, brain, intestine, kidney, placenta, spleen, stomach, and testes [96,98,99]. CES2 recognises substrates with a large alcohol group and a small acyl group; therefore, it is primarily responsible for the hydrolysis of prodrugs in which the alcohol group of an active pharmaceutical ingredient has been modified by a small acyl group [95,96]. Such modification enhances cell permeability, allowing these prodrugs to be easily taken up by enterocytes, where they may subsequently be hydrolysed intracellularly to release the active compound [95].

In contrast, CES1 predominantly hydrolyses prodrugs in which the carboxyl group of the active drug (a large acyl group) has been modified by a small alcohol group [98,100]. These prodrugs remain stable in the intestine but are extensively hydrolysed in the liver, where CES1 expression dominates. Although both CES1 and CES2 are present in the human liver, CES1 expression is significantly higher. CES1 is also the dominant isoform in the oesophagus, larynx, liver, lung, heart and trachea [96,98]. For a long time, it was thought that the active site structure of CES1 allows for greater “substrate promiscuity”—a term that refers to an enzyme ability to act on structurally distinct substrates [99,101]. However, the latest research shows that the selectivity of the CES isoforms is not only related to the molecular size of the alkyl or acyl groups of the substrates, but it is a more complex question, discussed in detail by Ribone et al. [101].

While there is plenty of information about the structure, active site, substrate preferences, and genetic polymorphisms of CESs, the question of which specific CES hydrolyses DMF has not yet been satisfactorily answered.

In the well-cited study by Werdenberg et al. [23], DMF was hydrolysed in the presence of both intestinal perfusate and intestinal homogenate, with the latter proving more effective. And as mentioned, the authors subjected DMF also to chamber transport studies and observed rapid passage of intact DMF through a Caco-2 cell monolayer, whereas its permeability through excised intestinal mucosa could not be measured due to quick and complete hydrolysis of DMF to MMF during transport.

In the search for the specific esterase responsible for DMF hydrolysis, Yang et al. recently demonstrated that among the two major CES groups, only CES1 was able to hydrolyse DMF in a time-dependent manner under conditions mimicking the intracellular environment, while almost no MMF production was observed with CES2 [24]. The same observation was mentioned by another research group [31]. This is, after all, in accordance with the enzymes chemical structural differences, active sites and spatial organisations [31,96,97,98].

An intriguing aspect of Yang’s and Werdenberg’s observations is that, at the time of Werdenberg’s study, the major intracellular esterase in Caco-2 cells was CES1, not CES2 [95,102] (authors’ note: nowadays, Caco-2 subclones with different esterase activity are also available, including CES2 [103]). In contrast, in the porcine intestine—specifically the jejunal part used by Werdenberg—there is no record of CES1 expression according to the Bgee database [104,105]. Based on this, observations by Werdenberg et al. should have yielded exactly the opposite result—yet this was not the case. Since then, accumulating evidence supporting the notion that DMF undergoes enzymatic hydrolysis within intestinal cells has been growing, despite the low CES1 and high CES2 activity in that region [97,102].

A recent publication by Bojanowski et al. [106] brought unexpected findings. The authors introduced a novel fumarate derivative, isosorbide di-(methylfumarate), which they considered a prodrug of DMF—even though this molecule, with a central isosorbide moiety, possesses two MMF residues. Since the compound is intended for topical use in psoriasis, where DMF has been shown to be harmful, and CES2 expression was previously observed in keratinocytes [107], the authors subjected both DMF and the new compound to in vitro test-tube incubation with CES2. Surprisingly, they observed complete hydrolysis of both substances within 60 min, making this research group, to our best knowledge, the first one to directly demonstrate CES2-mediated hydrolysis of DMF in vitro. Unfortunately, the authors did not specify the CES2 protein species origin or splicing variant.

On the other hand, Yang et al. [24], consistent with their own and others’ findings on CES1-mediated DMF hydrolysis, proposed that DMF is primarily hydrolysed in the liver (by hepatic CES1), and that hydrolysis in the gastrointestinal tract or other tissues is unlikely to play a significant role in the release of the active ingredient, MMF. This was surprising, because, in addition to observations by Werdenberg et al., no DMF was found in the portal vein of rats following direct intestinal administration of DMF and this finding was well-cited [22]. What was detected in the portal vein as early as 2 min after DMF injection, on the other hand, was MMF and a small fraction of breakdown products of GSH-DMF and GSH-MMF adducts. GSH-DMF-derived metabolites were found in higher concentrations exclusively in the intestinal tissue, indicating extensive reaction of DMF with GSH in the mucosa, followed by further degradation of these new adducts either in the portal vein blood or in earlier compartments [22]. This observation is particularly relevant to those proposing that no intact DMF reaches the liver.

But how does this translate to humans? A recent detailed comparative study of Serri et al. reported complete hydrolysis of DMF in ex vivo rat blood samples within seconds—even when cooled to 4 °C—accompanied by a corresponding increase in MMF concentrations [30]. DMF is known to undergo spontaneous hydrolysis at pH 7.4 (and above) even in the absence of enzymatic assistance, as shown previously [29]. However, in normal human serum at 37 °C, the rate of spontaneous DMF hydrolysis was much lower than what was observed in rat blood by Serri et al. (compare [29] and [30]). Yet, in addition to the sensitivity of the analytical method, which cannot be overlooked, there is another significant difference between human and rat blood. In rats, CES1 is secreted by the liver into the circulation in substantial amounts [108,109], which may affect the levels of DMF in the rat blood, whether ex vivo or in the portal vein [22,30]. Though low temperature is not optimal for enzyme activity, it cannot be ruled out. On the other hand, the presence of GSH-DMF and GSH-MMF conjugates accumulated in the rat intestinal tissue suggests that DMF hydrolysis occurs already within enterocytes [22], despite the fact that the CES isoenzymes expressed in rat intestine belong to the CES2 family [98]. In addition, only CES2 isoenzymes have been identified in the rat stomach as well [110].

Another layer of uncertainty arises from the fact that, while CES1 is present in human plasma only at almost undetectable levels, with its activity generally considered negligible [111,112], there are still reports of DMF hydrolysis in human serum and whole blood under experimental conditions [29,72]. Thus, the question remains not only whether intact DMF reaches the portal vein or the liver, but also which plasma hydrolase is responsible, as it is still unclear whether DMF can serve as a substrate for other circulating human esterases such as butyrylcholinesterase, paraoxonase, or albumin esterase [108].

Taken together, the question of such “familiar” esterase-mediated hydrolysis has become increasingly complex and resolving it is crucial for the proper interpretation of DMF- or MMF-related research—particularly when aiming to understand the full range of intracellular mechanisms underlying the clinical efficacy of either compound.

For, if CES1 is the enzyme responsible—and its expression was confirmed in numerous cell types [96,98,113]—how can we be sure that hydrolysis is not already occurring during in vitro incubation of cell cultures with DMF? Which of the observed intracellular effects and supposed mechanistic actions can then be undoubtedly attributed to DMF, and which of them can be initiated by MMF—or even by methanol or formaldehyde? In fact, Piroli et al. detected both DMF- and MMF-modified protein thiols after incubating ex vivo astrocytes and in vitro neurons with DMF, thereby confirming ongoing ester hydrolysis of DMF inside these cells during incubation [114].

**Figure 2:**
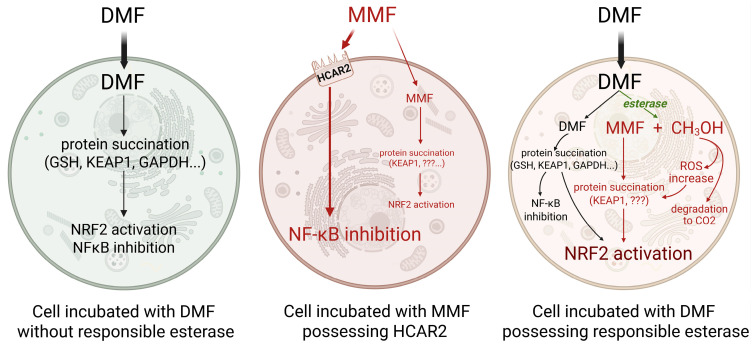
Simplified scheme comparing selected intercellular processes in the cells incubated with DMF or MMF. Cell possessing esterase for DMF hydrolysis is subjected to more effects due to MMF and methanol production. A small intercellular amount of less permeable MMF, followed by a lower effect, is depicted by small letters. Abbreviations: DMF—dimethyl fumarate, MMF—monomethyl fumarate, GSH—glutathione, KEAP1—Kelch-like ECH-associated protein 1, GAPDH—glyceraldehyde 3-phosphate dehydrogenase, NF-κB—nuclear factor κB, NRF2—nuclear factor erythroid 2-related factor 2, HCAR2—hydroxycarboxylic acid receptor 2. Created in BioRender. Kopincova, J. (2025) https://BioRender.com/zp4szt8, accessed on 18 November 2025.

It is important to note that if only certain in vitro-treated cells express CES able to hydrolyse intracellular DMF to MMF (and methanol—whose role is increasingly recognised), while other cells do not, the resulting intracellular mechanisms may differ substantially (Figure 2).

On the other hand, if CES2 is the responsible one and DMF is not detected in human plasma, an important question arises: is the extent of GSH-adduct formation within enterocytes so substantial that only the hydrolysed ester, i.e., MMF, is able to exit the cells? Or is a single passage through enterocytes sufficient to ensure complete enzymatic hydrolysis? Or are there any other enzymes acting? The apparent “loss” of a significant portion of DMF during its transition from the intestinal lumen to the bloodstream, as recently demonstrated [30,47], represents a compelling topic for further investigation.

## 7. Issue 4: Methanol—Reason for Gastrointestinal Events and Flushing, or More?

If intracellular hydrolysis of DMF proceeds in the gastric or intestinal mucosa or in some other cells, a molecule of methanol is also released alongside MMF formation.

To the best of the authors’ knowledge, based on a review of more than 500 relevant publications, methanol as a by-product of DMF hydrolysis was first mentioned in relation to gastrointestinal events in 2013 [35]. However, it had already been recognized and studied by Biogen Inc. during the FDA approval process for Tecfidera^®^ in 2012–2013 [20,21]. Since then, DMF-derived methanol was referenced only once, in a study focused on colon cell viability in relation to GSH depletion and increased ROS production [115]. Palte et al. [36] highlighted methanol and its metabolites as established gastrointestinal irritants in the context of the newly introduced diroximel fumarate. Unlike DMF, diroximel fumarate produces significantly less methanol during its metabolism (a 1:10 ratio relative to the active ingredient, compared to 1:1 in DMF). Palte’s publication prompted a noticeable increase in papers referencing methanol in the context of DMF hydrolysis and digestive side effects (e.g., [116,117,118]), including one that refers to methanol as the primary metabolite of DMF metabolism [116].

Though it may sound somewhat humorous at first, this perception of methanol might not be so far from the truth. If in vitro tissue cells incubated with DMF possess a CES capable of hydrolysing DMF upon its entry into the intracellular space, the fate of the absorbed DMF may be twofold: part of it likely undergoes thiol adduction, while another portion may be hydrolysed to MMF and methanol. The formation of GSH adducts, a process known to occur rapidly, lowers the availability of reduced GSH and disrupts the cellular redox balance [37,119]. But methanol does the same.

In the human body, the small amount of methanol generated from the hydrolysis of a standard therapeutic dose of DMF is likely to be efficiently metabolized by three distinct enzymatic systems. In the liver, the primary role is played by cytosolic alcohol dehydrogenase class I (ADH1), which oxidizes approximately 90% of methanol to formaldehyde while reducing NAD^+^ to NADH. Throughout the body, ten ADH isoforms, divided into five classes, are expressed [120].

In brain tissue which lacks ADH1, methanol is instead metabolized via the catalase-peroxidase system, which consumes H_2_O_2_, and by cytochrome P450 monooxygenases (particularly CYP2E1), whose activity generates reactive oxygen species (ROS) that, in turn, deplete reduced GSH levels [85,120,121]. This can be a significant challenge to newly introduced intranasal administration of DMF via nanoparticles, which facilitates nose-to-brain delivery without entering systemic circulation [30,47]. Bypassing first-pass hepatic metabolism shifts the entire burden of methanol release to the brain tissue, which could be detrimental if the dose is not properly titrated.

The above-mentioned three systems are widely expressed across human tissues, with some minor variances, and although their contribution to methanol metabolism differs by location, all ultimately convert methanol to formaldehyde and contribute to a reduction in the cell antioxidant capacity—either through depletion of oxidized cofactors, increased ROS production, or both. Notably, a decrease in the NAD^+^/NADH ratio inhibits xanthine dehydrogenase and promotes its conversion to xanthine oxidase, further generating superoxide anions [121,122].

Methanol-derived formaldehyde is subsequently oxidized in the cell to formic acid through three additional distinct pathways: cytochrome P450 monooxygenases, cytosolic and mitochondrial aldehyde dehydrogenase (ALDH) isoenzymes, and the primary formaldehyde-oxidizing enzyme gene encoding alcohol dehydrogenase 5 (ADH5), also known as ADH3 or formaldehyde dehydrogenase (FDH) [120]. Since formaldehyde is more toxic to cells than methanol itself, FDH is active in nearly all human tissues (according to the Bgee database, FDH is expressed in 211 tissue types [104,123]). This GSH-dependent enzyme initially allows formaldehyde to bind spontaneously to GSH, forming S-formylglutathione without enzymatic catalysis. S-formylglutathione then serves as the substrate for FDH, which converts it into formic acid while consuming NAD^+^ [120]. Formic acid dissociates into formate and hydrogen ions [122], and formate is either excreted by urine or further oxidised to CO_2_ and eliminated via exhalation [120].

Elimination of formaldehyde is usually rapid, taking only minutes; however, this depends on the enzymatic profile of the cell [120,122]. In contrast, formate is eliminated from the body more slowly, primarily by oxidation into CO_2_—a process most intensive in the liver. The hepatic content of tetrahydrofolate (H_4_-folate), required for this reaction, is considered rate-limiting [122]. Accumulation of formate in the body is believed to be the primary cause of the clinical manifestations of methanol poisoning, mainly due to metabolic acidosis, increased ROS generation, and inhibition of mitochondrial cytochrome oxidases [120,122].

Though the enzymatic apparatus for methanol elimination is extensive, even the small amount of methanol released from a standard dose of DMF—or more precisely, its downstream metabolites—can be potent enough to trigger clinically significant episodes of nausea, vomiting, epigastric pain, and flushing, sometimes so severe that they lead to treatment discontinuation or even more serious outcomes [36,67,89,124]. Moreover, differences in genetic background, such as polymorphisms in ALDH alleles, significantly influence the rate of methanol metabolism and may result in formaldehyde accumulation in tissues, accompanied by, for example, pronounced facial flushing [85,125]. Notably, skin flushing has been, for more than 40 years, associated with the accumulation of alcohol-derived aldehydes, including formaldehyde [126,127,128,129]. However, in association with DMF intake, other triggers of flushing besides methanol have been reported—particularly vasoactive prostaglandins induced by HCAR2 activation [17,18,25]. These cannot be ruled out, particularly because flushing also occurs during MMF and diroximel-fumarate treatment, where methanol production is low [48,116,118,130].

However, during in vitro incubation of CES-expressing tissue cultures with DMF, cells lack the metabolic support of a high-capacity organ such as the liver for efficient methanol clearance and their available intrinsic enzymatic profile may slow down elimination processes. The same situation occurs after direct nose-to-brain DMF administration [30,47]. Formaldehyde, when present, readily reacts with amino and sulfhydryl groups of many proteins, peptides, and nucleic acids, including GSH. Between 50% and 80% of endogenous formaldehyde in the body is bound within GSH-containing complexes. Since GSH also serves as a cofactor for FDH, a drop in GSH levels not only reduces formaldehyde detoxification but also increases its toxicity [122]. The affinity of DMF for GSH, due to its unsaturated double bond, is high, and adducts are formed within minutes [37]; however, DMF-derived methanol—via its own metabolism—may also contribute to GSH depletion, ROS production and other alterations which can then affect cellular responses. As a consequence of GSH depletion and increased ROS production, NRF2 may become activated even without direct binding of DMF or MMF to its regulatory factor KEAP1. In fact, some authors consider this mechanism—i.e., glutathione depletion—to be a primary pathway of the biological action of fumarate esters [44,65,119].

This opens another important question—what are the most important intracellular molecular mechanisms triggered by DMF or MMF involved in their therapeutic actions?

## 8. DMF—Universal Repurposing?

DMF is recognised as a molecule with pleiotropic effects, and evidence regarding its potential mechanisms of action is rapidly accumulating.

The commonly accepted pathways associated with DMF therapeutic effects include particularly: (1) Succination of cysteine residues on proteins (predominantly related to GSH, but other important proteins, e.g., hepatocyte nuclear factor 1β, DNA-dependent protein kinase or glyceraldehyde 3-phosphate dehydrogenase, are also mentioned) [26,44,131]; (2) Activation of NRF2-dependent antioxidant pathway, which has been confirmed by studies on NRF2 knockout mice on the in vitro effect of MMF, and in vivo effect of DMF, mediated by succination of KEAP1 cysteine, GSH depletion or other mechanisms [62,68,132]; (3) MMF-mediated HCAR2 activation associated with microglial and phospholipase C activation, increase in prostaglandin formation and others [18,45,93]; (4) Inhibition of NF-κB, by distinct actions, including also HCAR2 activation, with various consequences [5,6,45,133]. The mechanisms of anti-inflammatory actions of fumarates have been detailed in several recent comprehensive reviews [44,45,134]; (5) Suppression of NLRP3 inflammasome activation and concomitant pathways, including pyroptosis [7,8,9,10,135,136], reviewed in detail in [137,138].

Novel pathways are being identified, involving intracellular factors such as peroxisome proliferator-activated receptor gamma (PPAR-γ), sirtuin 1, a novel ferroptosis amplifier stimulator of interferon genes (STING), JAK2–STAT3 pathway and many others [139,140,141].

However, much of our current knowledge about the mechanistic actions of DMF comes from in vitro cell culture studies, apart from the complexity of the whole-body system, and often without considering possible hydrolysis of DMF during in vitro incubation with the independent intracellular effects of possibly co-produced methanol.

In many cases, even the authors themselves may not always be able to determine whether the reported results are due to the action of DMF and/or MMF, or their other metabolites, and whether they occur only in vitro or are also relevant in vivo.

Despite above mentioned issues, the therapeutic potential of DMF, and monomethyl derivates, goes far beyond multiple sclerosis and psoriasis. DMF is investigated on clinical or experimental level for the repurposing for remarkably broad range of disorders. The main attention is given to neural and neurodegenerative diseases, including adrenomyeloneuropathy (EUCT 2023-506795-27-00), age-related macular degeneration (NCT04292080, EUCT2024-510741-33-00) [132], Alzheimer’s disease (NCT06850597; EUCT 2024-517214-16-00) and Parkinson’s disease [142,143]. Experimental investigations are conducted in the area of various eye pathologies [144], cardiovascular diseases [66,145] and gastrointestinal and liver diseases [9,146], and many others of all kinds, like muscular dystrophy [147], renal cell carcinoma [131], diabetic erectile dysfunction [148], lung inflammation [149] and so on. The vast majority of diseases that have been treated with DMF are rooted in oxidative damage and the inflammatory cascades—which makes DMF, with all its currently recognized effects, a logical choice. Moreover, as pharmacological activation of NRF2 has also been shown to delay senescence, the benefits of DMF for healthy ageing may still remain to be uncovered [150].

On the other hand, enthusiasm for the potential therapeutic repurposing of fumarates across a broad spectrum of diseases should be tempered by awareness of the possible detrimental consequences of sustained constitutive NRF2 activation, which can promote tumorigenesis, tumour survival and metastasis, or end up in the induction of damaging reductive stress [66,151,152]. Moreover, through the years, significant number of serious adverse events was recorded by the FDA following DMF therapy, including haemorrhage, ulceration, colitis or enterocolitis and severe leukopenia [124].

## 9. Conclusions

This review highlighted several unresolved issues related to pharmacology of DMF and its metabolites that have not yet been fully elucidated. It seems that a lot is known about what happens after DMF enters cells in vitro. However, relatively little is known about what happens before DMF enters target cells after oral administration, and it is not even clear whether DMF per se enters certain cells at all. Unresolved issues likewise include the question of which intracellular mechanisms—or their combinations—are the most important in the treatment of each specific disease.

Thus, there is much room for further preclinical research, which will bring a more comprehensive understanding of the pharmacokinetics, pharmacodynamics and mechanisms of action of individual fumarates and their derivatives, and their use for the treatment of non-malignant non-communicable diseases and healthy ageing.

## Figures and Tables

**Table 1:** Comparison of selected characteristics of dimethyl fumarate and monomethyl fumarate related to pharmacokinetics and pharmacodynamics.

Parameter/Process	DMF	MMF	References
Polarity	Non-polar	Polar	[28]
Water solubility	1.6 mg/mL	20 mg/mL	[28]
Membrane permeability	Higher (~10-times)	Lower	[23]
Spontaneous hydrolysis (pH ≥ 7.4)	Higher	Much lower	[29,30]
Susceptibility to CES hydrolysis	Yes	Poor	[30,31]
Michael addition	Higher rate	Lower rate	[32,33,34]
Methanol release	Yes	No	[35,36]
HCAR2 agonist	No	Yes	[16,17]
Cellular GSH depletion	Higher (~30-times)	Lower	[37]
Acute oral toxicity (rat)	LD_50_ > 2.63 g/kg	LD_50_ > 11.5 g/kg	[38,39]

Abbreviations: CES—Carboxylesterase, DMF—Dimethyl fumarate, GSH—Glutathione, HCAR2—Hydroxycarboxylic acid receptor 2, LD_50_—Median lethal dose, MMF—Monomethyl fumarate.

**Table 2:** Approved drugs for human use containing dimethyl fumarate, monomethyl fumarate or pro-drugs of monomethyl fumarate.

Drug	Approval	Dosage Forms and Strengths	Dosing	References
Fumaderm^®^ (DMF, MEF salts)	BfArM (1994); discontinued in 2024	Gastro-resistant tablets: 120 mg DMF and 95 mg MEF salts	1 or 2 tablets up to 3 times a day (max. 720 mg of DMF daily), orally	[52,53]
Tecfidera^®^ (DMF)	FDA (2013) EMA (2014)	Delayed-release capsules: 120 mg or 240 mg	Starting dose: 120 mg capsule twice a day, orally Maintenance dose: 240 mg capsule twice a day, orally	[20,54]
Skilarence^®^ (DMF)	EMA (2017)	Gastro-resistant tablets: 120 mg	1 or 2 tablets up to 3 times a day (max. 720 mg daily), orally	[38]
Vumerity^®^ (Diroximel fumarate)	FDA (2019) EMA (2021)	Delayed-release capsules: 231 mg	1 or 2 tablets twice a day (924 mg daily), orally	[55,56]
Bafiertam™ (MMF)	FDA (2020)	Delayed-release capsules: 95 mg	Starting dose: 95 mg twice a day, orally Maintenance dose: 190 mg (two 95 mg capsules) twice a day, orally	[57]
Riulvy (Tegomil fumarate)	EMA (2025)	Gastro-resistant hard capsules: 174.2 mg or 348.4 mg	Starting dose: 174.2 mg capsule once daily, Maintenance dose: 348 mg capsule twice daily, orally	[49]
Tepilamide fumarate	Phase IIb clinical study NCT03421197	Extended-release tablets: 400 mg or 600 mg	400 mg tablet once or twice daily, 600 mg tablet twice daily, orally	[50]

Abbreviations: BfArM—German Federal Institute for Drugs and Medical Devices; DMF—Dimethyl fumarate, EMA—European Medicines Agency, FDA—U.S. Food and Drug Administration, MEF—Monoethyl fumarate, MMF—Monomethyl fumarate.

## Data Availability

Not applicable.

## Abbreviations

The following abbreviations are used in this manuscript:

ADH1	Alcohol dehydrogenase class I
ADH5	Alcohol dehydrogenase 5
ALDH	Aldehyde dehydrogenase
ARE	Antioxidant-responsive elements
AUC	Area under the curve
BfArM	German Federal Institute for Drugs and Medical Devices
CES1	Carboxylesterase-1
CES2	Carboxylesterase-2
CESs	Carboxylesterases
*C_max_*	Maximum plasma concentration
DMF	Dimethyl fumarate
EMA	European medicines agency
FDAA	U.S. Food and Drug Administration
FDH	Formaldehyde dehydrogenase
GAPDH	Glyceraldehyde 3-phosphate dehydrogenase
GSH	Glutathione
HCAR2	Hydroxycarboxylic acid receptor 2
HIF-1α	Hypoxia-inducible factor 1α
HO-1	Heme oxygenase-1
IUPAC	International union of pure and applied chemistry
KEAP1	Kelch-like ECH-associated protein 1
LD_50_	Median lethal dose
MEF	Monoethyl fumarate
MMF	Monomethyl fumarate
MS	Multiple sclerosis
NF-κB	Nuclear factor κb
NRF2	Nuclear factor erythroid 2-related factor 2
PD	Pharmacodynamics
PK	Pharmacokinetics
ROS	Reactive oxygen species
*t_max_*	time to maximum plasma concentration
